# Screening for adult attention deficit and hyperactivity disorder among military parents: A pilot study

**DOI:** 10.1192/j.eurpsy.2021.565

**Published:** 2021-08-13

**Authors:** A. Ben Othman, H. Slama, I. Soualmia, A. Oumaya

**Affiliations:** 1 Child And Adolescent Psychiatry, Military Hospital of Instruction of Tunis, Tunis, Tunisia; 2 Psychiatry, Military Hospital of Instruction of Tunis, Tunis, Tunisia

**Keywords:** Adult ADHD, Child ADHD, military

## Abstract

**Introduction:**

The prevalence of adult Attention Deficit and Hyperactivity Disorder (ADHD) has been investigated in the general population by multiple studies. However, few studies have focused on identifying its prevalence in the military population, particularly among military parents of children with ADHD.

**Objectives:**

The aim of our study was to screen for adult ADHD among military parents of ADHD children followed-up at the child and adolescent psychiatry department in the Military Hospital of Instruction of Tunis, Tunisia.

**Methods:**

This prospective study was carried among military parents (one or both parents belonging to the national army) of ADHD children. Children were diagnosed with ADHD based on the 5^th^ Diagnostic and Statistical Manual ADHD criteria and the Conners Comprehensive Behavior Rating Scale. Whereas adult ADHD was screened for using the Adult ADHD Self-Report Scale-V1.1.

**Results:**

Fifteen children and twenty-nine parents were included in the study: sixteen of the parents were military members and thirteen were civilian spouses. Eight (50%) of the sixteen military parents, and four (30,7%) of the civilian spouses were screened positive for ADHD. Whereas 73% of these children had at least one parent screened positive for ADHD, and 53% had at least one military parent screened positive for ADHD. These results suggest a high prevalence of adult ADHD among this population.

**Conclusions:**

ADHD occurs in childhood and may persist into adulthood. The findings of this study indicate that ADHD symptoms are not limited to the youth and are common in military population. Implications on screening, management, preventive measures and research should be discussed.

